# Mulberroside A: A Multi-Target Neuroprotective Agent in Alzheimer’s Disease via Cholinergic Restoration and PI3K/AKT Pathway Activation

**DOI:** 10.3390/biology14091114

**Published:** 2025-08-22

**Authors:** Jin Li, Jiawen Wang, Yaodong Li, Jingyi Guo, Ziliang Jin, Shourong Qiao, Yunxia Zhang, Guoyin Li, Huazhen Liu, Changjing Wu

**Affiliations:** 1 Department of Basic Veterinary Medicine, College of Animal Science and Veterinary Medicine, Huazhong Agricultural University, Wuhan 430070, China; lijin0213@webmail.hzau.edu.cn; 2 Institute of Translational Medicine, College of Life Science and Agronomy, Zhoukou Normal University, Zhoukou 466001, China; 15537358569@163.com (J.W.); lyd1572023@163.com (Y.L.); 19903948973@163.com (J.G.); 19839031026@163.com (Z.J.); 13137420683@163.com (S.Q.); yunxiaflight@163.com (Y.Z.); ligy@zknu.edu.cn (G.L.); 3 Fuxi Laboratory, Zhoukou Normal University, Zhoukou 466001, China

**Keywords:** Mulberroside A, Alzheimer’s disease, neuroprotection, cholinergic system, cognitive impairment, PI3K/AKT signaling

## Abstract

Alzheimer’s disease (AD) is a progressive neurodegenerative disorder and the leading cause of dementia, with current therapies offering only limited symptomatic relief and lacking disease-modifying efficacy. Addressing this critical therapeutic gap, natural multi-target compounds like mulberroside A (MsA)—a bioactive glycoside from *Morus alba* L.—present promising alternatives. This study investigated MsA’s neuroprotective potential using scopolamine-induced AD-like mice and N2a/APP695swe cells. In vivo, MsA significantly ameliorated cognitive deficits and neuronal loss, concurrently enhancing cholinergic neurotransmission through increased acetylcholine levels and inhibited acetylcholinesterase (AChE)/butyrylcholinesterase (BChE) activities. MsA also upregulated neurotrophic factors (BDNF, CREB) in critical brain regions. In vitro, MsA restored cholinergic function, mitigated oxidative stress, and crucially reduced amyloid-β (Aβ) production by dual regulation of APP processing: promoting the non-amyloidogenic pathway via ADAM10 upregulation and inhibiting the amyloidogenic pathway via suppression of BACE1 and γ-secretase components. Mechanistically, these multi-target benefits were mediated by MsA’s activation of the PI3K/AKT pathway, which triggered downstream inhibitory phosphorylation of GSK3β—directly reduced tau hyperphosphorylation—and activation of CREB/BDNF signaling. Collectively, our findings demonstrate that MsA confers comprehensive neuroprotection against AD pathology by simultaneously targeting cholinergic dysfunction, oxidative stress, Aβ accumulation, tau phosphorylation, and impaired neurotrophic signaling, highlighting its strong therapeutic candidacy.

## Simple Summary

Alzheimer’s disease is a severe, gradually worsening brain disorder leading to memory loss and dementia, with no effective treatments currently available. In this study, we investigated a natural compound called mulberroside A, derived from the mulberry plant, to determine its potential protective effects against Alzheimer’s disease. Utilizing a mouse model and human brain cells, we discovered that mulberroside A enhanced memory and decreased brain cell damage. Additionally, it increased the levels of crucial brain chemicals, facilitating better communication between nerve cells. Moreover, it alleviated harmful stress in the brain and reduced the formation of two hallmark pathologies: toxic tau protein tangles and amyloid plaques. The compound significantly decreased phosphorylated tau—a key driver of neuronal degeneration—while simultaneously inhibiting amyloid plaque formation. Mulberroside A operated by activating specific neuroprotective pathways that promote brain health, including those that encourage nerve growth. These findings indicate it could serve as a promising new treatment to slow or prevent Alzheimer’s disease by targeting multiple disease mechanisms.

## 1. Introduction

Alzheimer’s disease (AD) is a progressive neurodegenerative disorder and the leading cause of dementia worldwide, marked by significant deficits in memory, learning, and executive function [1]. While the exact etiology of this disease remains unclear, extensive research has highlighted a complex pathology involving cholinergic dysfunction, the abnormal accumulation of amyloid-beta (Aβ) peptides, neurofibrillary tangles composed of hyperphosphorylated tau protein, and increased oxidative stress [2,3,4]. The cholinergic hypothesis posits that the degeneration of basal forebrain cholinergic neurons leads to decreased acetylcholine (ACh) levels in cortical regions, a condition further intensified by increased activity of cholinesterases, notably acetylcholinesterase (AChE) and butyrylcholinesterase (BChE) [5,6]. Concurrently, toxic Aβ peptides result from the sequential cleavage of amyloid precursor protein (APP) by β-secretase (BACE1) and γ-secretase complexes, while activation of α-secretase (ADAM10) promotes a protective, non-amyloidogenic pathway [7,8]. Critically, aberrant tau hyperphosphorylation leads to neurofibrillary tangle formation, disrupting microtubule stability and impairing neuronal transport [9]. Additionally, oxidative damage to neurons and impaired neuronal survival mediated by transcription factors such as brain-derived neurotrophic factor (BDNF) and cAMP response element-binding protein (CREB) significantly contribute to synaptic dysfunction [10,11,12]. Current therapeutic options, such as cholinesterase inhibitors, NMDA receptor antagonists, and recent anti-Aβ monoclonal antibodies, provide only modest clinical improvements, along with significant side effects and uncertain long-term outcomes efficacy [13,14,15]. The significant unmet medical need highlights the need for innovative, multi-target therapies that can simultaneously target the interconnected pathological mechanisms of AD.

Natural bioactive compounds offer potential alternative treatments for AD, demonstrating diverse biological activities, significant efficacy, and low toxicity in preclinical studies [16,17]. Among these, mulberroside A (MsA), a glycosylated stilbene from *Morus alba* L., has garnered attention for its antioxidative, anti-inflammatory, and neuroprotective capacities across disease models [18,19,20,21]. Previous studies indicate that *Morus alba* L. root cortex extract inhibits critical enzymes involved in cholinergic dysfunction (AChE and BChE) and amyloidogenesis (BACE1), reduces malondialdehyde (MDA) levels, attenuates tau pathology, and enhances antioxidant enzyme activity, thus suggesting comprehensive therapeutic benefits for AD. Notably, these benefits translated to improved cognitive performance, including enhanced spatial memory in metabolic impairment models [22,23,24]. Nevertheless, the exact mechanisms—particularly regarding cholinergic transmission, Aβ regulation, tau pathology, and neurotrophic signaling—remain incompletely characterized. Given the pivotal role of the PI3K/Akt signaling pathway in AD pathogenesis—through regulation of GSK3β-mediated amyloidogenic processing (suppression of α-secretase ADAM10 and promotion of γ-secretase activity) [25,26], tau phosphorylation dynamics [27], and neurotrophic support via CREB-dependent BDNF expression [28,29]—combined with evidence that mulberry-derived compounds activate Akt/GSK3β signaling [30], we hypothesized that MsA could provide effective multi-target neuroprotection by simultaneously improving cholinergic dysfunction, reducing amyloidogenic pathways, decreasing oxidative stress, inhibiting tau phosphorylation, and enhancing neurotrophic signaling through modulation of the PI3K/Akt pathway.

To validate this hypothesis, complementary in vivo and in vitro AD models were employed to systematically assess MsA’s neuroprotective properties. For the in vivo component, scopolamine-induced cognitive impairment—characterized by muscarinic receptor blockade, elevated hippocampal and cortical AChE activity, and reduced ACh availability—was selected to replicate central cholinergic deficiency, a fundamental AD pathological feature. This established pharmacological paradigm effectively mimics early AD symptoms including learning and memory deficits. The model’s translational validity is further reinforced through mechanistic congruence with Donepezil (our positive control), a first-line AD therapeutic exerting efficacy via AChE inhibition. Behavioral assessments (Novel Object Recognition, Morris Water Maze) quantified cognitive impairment in scopolamine-treated mice, complemented by histological (Nissl staining, BDNF IHC), biochemical (ACh, AChE, BChE), and molecular (BDNF, CREB) analyses of hippocampal and cortical tissues. In vitro, N2a/APP695swe cells elucidated MsA’s protective mechanisms against cholinergic dysfunction, oxidative stress, tau phosphorylation, and APP processing alterations. Mechanistic investigations focused on MsA’s modulation of the PI3K/Akt/GSK3β/CREB pathway—critical for governing tau metabolism, neurotrophic signaling, and neuronal viability. This integrated strategy provides comprehensive preclinical validation of MsA’s multi-target therapeutic potential for AD.

## 2. Materials and Methods

### 2.1. Scopolamine-Induced Mouse Model

**Figure 1:**
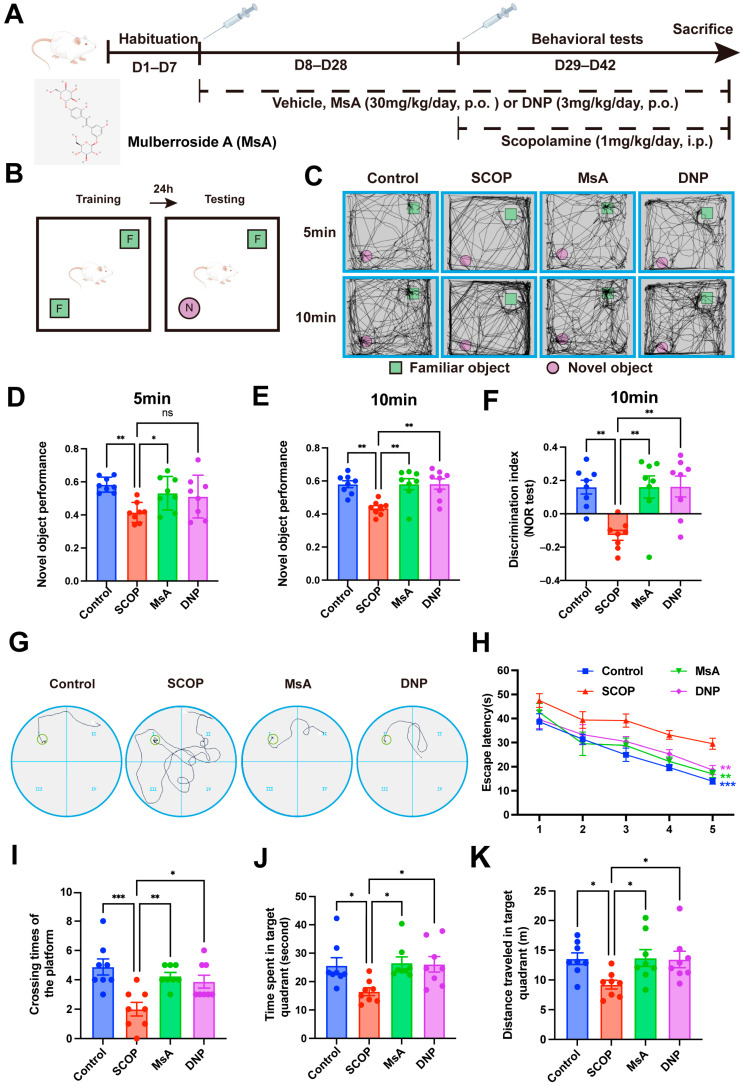
MsA alleviates cognitive impairments in scopolamine-induced mice. (**A**) Schematic timeline of the experimental protocol. (**B**–**F**) NOR test, schematic diagram of the NOR test (**B**), representative movement tracking images of mice exploring familiar (**F**) and novel (N) objects at 5 min and 10 min (**C**), quantification of the performance investigating the novel object at 5 min and 10 min (**D**,**E**) and discrimination index (DI) at 10 min (**F**). (**G**–**K**) MWM test, representative swimming paths of mice on the fifth day of the training phase (**G**) and escape latency during the 5-day training trials (**H**), crossing times of the platform (**I**), time spent in target quadrant (**J**), and distance traveled in target quadrant (**K**) during the probe trial (Supplementary Material Section S1.1). Data are expressed as mean ± SEM (*n* = 8 animals per group). * *p* < 0.05, ** *p* < 0.01, *** *p* < 0.001 vs. SCOP group. ns, no significance.

Thirty-two male ICR mice (4–6 weeks old) were procured from Nanjing University’s Model Animal Research Centre (Nanjing, China). Animals were group-housed (4/cage) in standard conditions: 12 h light/dark cycle, 23 ± 2 °C ambient temperature, with ad libitum access to food and water. Following 7-day acclimatization, mice underwent random allocation to four experimental cohorts: control (vehicle), scopolamine (SCOP) (Aladdin, Shanghai, China, CAS: 6533-68-2), SCOP + mulberroside A (MsA, 30 mg/kg/day, p.o.) (Aladdin, Shanghai, China, CAS:102841-42-9, purity exceeding 98%), and SCOP + Donepezil (DNP, 3 mg/kg/day, p.o.) (Targetmol, Shanghai, China, CAS: 120011-70-3). From days 8–28, oral treatments were administered daily. Cognitive impairment was subsequently induced in the SCOP groups via intraperitoneal scopolamine injections (1 mg/kg/day, days 29–42), while the controls received saline vehicles. Behavioral testing commenced with 30 min post-injection during this phase, with MsA/DNP pretreatment continuing through day 42. On day 42, all mice were euthanized for subsequent biochemical and histological analyses. Figure 1A illustrates the schematic timeline of the animal experimental protocol. All protocols were approved by Zhoukou Normal University’s Animal Welfare Committee (ZKNU-20240132; 3 January 2024), and all procedures adhered to ethical guidelines for the care and use of laboratory animals.

### 2.2. New Object Recognition Test

The novel object recognition test (NOR) was performed to evaluate recognition memory in ICR mice after administering scopolamine. The experiment was conducted in a controlled open-field arena measuring 40 cm on each side. In the training phase, individual mice were positioned in an arena featuring two identical cube-shaped objects (F, 4 cm in diameter) arranged symmetrically, allowing free exploration for 10 min. Following a 24 h postponement in the testing phase, a familiar object was substituted with a cylindrical object (N, 4 cm in both diameter and height), and the exploration was recorded for 5 and 10 min. Exploration was operationally defined as sniffing, touching, or nose orientation toward an object within 2 cm. Novel object performance was calculated as N/(N + F), where N is the time spent exploring the novel object and F is the time exploring the familiar one. The discrimination index (DI) was determined using (N − F)/(N + F). Locomotor activity was tracked and analyzed with an automatic path-tracking system (Zhongshidichuang Sci. and Tech., Beijing, China). After each trial, the arena and objects were sanitized with 75% ethanol to remove residual odors.

### 2.3. Morris Water Maze Test

The Morris Water Maze (MWM) test was utilized to assess spatial learning and memory in mice, employing the DMS-2 system developed by the Chinese Academy of Medical Sciences in Beijing, China. The water maze comprised a circular pool segmented into four quadrants, with a concealed platform located beneath the surface in one of the quadrants. On the initial day, referred to as the habituation phase, the mice were positioned in the third quadrant facing the pool wall and permitted to swim freely for a duration of 60 s. If the platform was not located within the designated time, participants were directed to it and permitted to remain for 10 s. From days one to five of the training phase, mice were introduced into the water from various quadrants in a randomized order, with one trial conducted per quadrant each day. The latency for escape to the concealed platform was quantified. If a mouse failed to find the platform within 60 s, it was guided to the platform, and the latency was noted as 60 s. On day six, the platform was removed for the probe test, and mice were introduced into the pool from the quadrant opposite the previous platform location. The duration in the target quadrant, the count of platform crossings, and the distance traveled in the target quadrant during a 60 s interval were recorded. Behavioral data were systematically recorded and analyzed with the DMS-2 software system.

### 2.4. Nissl Staining

Coronal brain tissue sections were prepared according to standard procedures. After dewaxing, the sections underwent staining with Nissl stain (Servicebio, Wuhan, China, C0117) at room temperature for a duration of 10 min. Following staining, the sections were rinsed with distilled water to remove the dye, dehydrated using graded alcohol, and cleared with xylene (Aladdin, Shanghai, China, CAS: 1330-20-7). The sections were then embedded in neutral resin for final sealing. The regions were observed under a light microscope (Olympus, Tokyo, Japan), focusing particularly on hippocampal subregions (CA1, CA3, and DG) and cortical areas. Three mouse samples from each group were analyzed to quantify the number of healthy neurons.

### 2.5. Biochemical Analysis

Cholinergic function and oxidative stress-related biochemical parameters were evaluated using commercial assay kits, following the manufacturers’ guidelines. The concentrations of ACh and the activities of AChE and BChE were evaluated in N2a/APP695swe cells, as well as in mouse hippocampal and cortical tissues. ACh levels were quantified using the Acetylcholine Assay Kit (Nanjing Jiancheng, Nanjing, China, A105-1-1). AChE and BChE activities were assessed with the Acetylcholinesterase Assay Kit (Nanjing Jiancheng, Nanjing, China, A024-1-1) and Butyrylcholinesterase Assay Kit (Nanjing Jiancheng, Nanjing, China, A025-1-1), respectively. Lipid peroxidation was assessed through the measurement of MDA levels in cells utilizing the MDA Assay Kit (Solarbio, Beijing, China, BC0025). Measurements were conducted using a microplate reader (Bio-Rad, Hercules, CA, USA) according to the suppliers’ protocols.

### 2.6. Immunohistochemistry

Immunohistochemical analysis for BDNF expression in the hippocampus was performed as previously outlined [31]. Tissue sections underwent deparaffinization and rehydration, followed by the quenching of endogenous peroxidase activity using 0.3% hydrogen peroxide (Aladdin, Shanghai, China, CAS: 7722-84-1) in methanol. Antigen retrieval was achieved by heating the slides in citrate buffer (pH 6.0) (Servicebio, Wuhan, China, G1219) and then blocking non-specific binding with 5% goat serum (Servicebio, Wuhan, China, G1208) for 1 h at 37 °C. The sections were incubated overnight at 4 °C with a rabbit anti-BDNF primary antibody (Servicebio, Wuhan, China, GB11559, 1:200). Following PBS washing, the sections underwent incubation with biotinylated secondary antibodies (Vector Laboratories, Burlingame, CA, USA, AB_2687893, 1:400) for 30 min. Visualization of the antigen–antibody complex was achieved using the Vectastain ABC kit (Vector Laboratories, AB_2336382) and diaminobenzidine (DAB) (Servicebio, Wuhan, China, G1212). Finally, the slides were dehydrated, cleared, and mounted, and BDNF-positive neurons were quantified in the hippocampal CA3 and DG regions using an Olympus microscope equipped with a Canon camera.

### 2.7. Cell Culture and Treatment Procedures

N2a/APP695swe cells with stable expression of the human APP695swe mutation were obtained from Professor Yunwu Zhang at Xiamen University, Fujian, China. Cells were cultured in high-glucose Dulbecco’s Modified Eagle Medium (DMEM, Waltham, MA, USA) with 10% fetal bovine serum (FBS) (Gibco, Melbourne, Australia), along with 100 U/mL penicillin and 100 μg/mL streptomycin mixed liquid (Servicebio, Wuhan, China, G4003). To maintain stable expression of the APP695swe gene, the culture medium was additionally supplemented with 200 μg/mL of G418 (Amresco, Solon, OH, USA). Cells were incubated at 37 °C in a humidified atmosphere containing 5% CO_2_. The culture medium was altered, and the cells were passaged every 2 to 3 days when they reached 80% confluence. In the MsA treatments, MsA was solubilized in phosphate-buffered saline (PBS) (Servicebio, Wuhan, China, G4207), which acted as the vehicle control.

### 2.8. Cell Viability Assay

Cell viability was assessed using the Cell Counting Kit-8 (CCK-8) assay (Solarbio, China, Cat: CA1210) according to the manufacturer’s guidelines. N2a/APP695swe cells were seeded in 96-well plates (Servicebio, Wuhan, China, CCP-96H) at a density of 10,000 cells per well and incubated overnight in a humidified environment at 37 °C with 5% CO_2_ facilitate adherence. On the subsequent day, the culture medium was substituted with fresh medium containing different concentrations of MsA (1, 5, 10, 25, 50, and 100 μM) or PBS as a control. After a 24 h incubation, the treatment medium was removed, and the cells were rinsed twice with PBS. Then, 100 μL of fresh culture medium, along with 10 μL of CCK-8 reagent (Beyotime, Shanghai, China, C0038), was introduced to each well. The cells underwent an additional incubation period of 1 h at 37 °C. Absorbance at 450 nm was quantified utilizing an automated microtiter plate reader. Relative cell viability was assessed by normalizing the absorbance of treated cells to against that of the vehicle control group.

### 2.9. ROS Levels Assessment

The intracellular ROS levels in N2a/APP695swe cells were measured using the fluorescent probe DCFH-DA from a Reactive Oxygen Species Assay Kit (Beyotime, Shanghai, China, S0033). Cells were grown in confocal dishes (Servicebio, Wuhan, China, WG801001) and exposed to MsA at 25 μM and 50 μM for 24 h, with PBS serving as the control. After incubation, cells were rinsed with PBS and then treated with 10 μM DCFH-DA at 37 °C in the dark for 30 min, followed by three PBS washes. Fluorescence images were acquired via confocal laser scanning microscopy (Zeiss, Oberkochen, Germany). Fluorescence images were captured with confocal laser scanning microscopy (Zeiss, Oberkochen, Germany, LSM800), employing an excitation wavelength of 488 nm and an emission wavelength of 525 nm. Relative fluorescence intensity was quantified using ImageJ software (version 1.54f) and normalized to the control group.

### 2.10. Aβ1–42 ELISA Assay

The levels of Aβ1–42 in N2a/APP695swe cell lysates were determined with an ELISA Kit (R&D Systems, Minneapolis, MN, USA, DAB142), following the manufacturer’s instructions. Standards and samples’ absorbance was measured at 450 nm using a microplate reader. Aβ1–42 levels in the samples were measured using a standard curve created with the four-parameter logistic model, and the outcomes were normalized to the control group.

### 2.11. Quantitative Real-Time PCR

RNA extraction was performed on N2a/APP695swe cells and mouse hippocampal and cortical tissues utilizing Trizol reagent (Beyotime, Shanghai, China, R0016), following the manufacturer’s protocol. The RNA concentration and purity were measured with a NanoDrop 2000 spectrophotometer (Thermo Fisher Scientific, Waltham, MA, USA), while RNA integrity was checked using agarose gel electrophoresis, then followed by reverse transcription into cDNA utilizing the reverse transcription kit (Servicebio, Wuhan, China, G3337). Quantitative real-time PCR (qRT-PCR) was conducted using SYBR Green Realtime PCR Master Mix (Sangon Biotech, Shanghai, China, B690016) on a LightCycler system (Roche, Basel, Switzerland), following the manufacturer’s instructions. *GAPDH* served as an internal reference gene. Relative mRNA expression levels of *BACE1, ADAM10, PSEN1*, and *Nicastrin* in cells, as well as *BDNF* and *CREB* in hippocampal and cortical tissues, were calculated using the 2^−ΔΔCt^ method. Primer sequences are listed in Table 1.

### 2.12. Western Blotting Analysis

N2a/APP695swe cells were lysed in RIPA buffer (Servicebio, Wuhan, China, G2002) supplemented with protease and phosphatase inhibitors, and total protein quantities were quantified using a BCA Protein Assay Kit (Beyotime, Shanghai, China, P0010). Identical quantities of protein were resolved using 10% SDS-PAGE and subsequently transferred to nitrocellulose membranes (Millipore, Billerica, MA, USA). Membranes were incubated with 5% non-fat milk in TBST (Servicebio, Wuhan, China, G2150) for 1.5 h at ambient temperature, followed by overnight incubation at 4 °C with primary antibodies. Following washing, HRP-conjugated secondary antibodies were administered for one hour at ambient temperature. Bands were identified with ECL detection reagents (Bio-Rad, Hercules, CA, USA), and densitometric analysis was conducted with ImageJ. The primary antibodies included APP (Proteintech, Wuhan, China, 27320-1-AP, 1:1000), BACE1 (Sangon Biotech, Shanghai, China, D120305, 1:1000), ADAM10 (Servicebio, Wuhan, China, GB11318, 1:500), p-PI3K (Cell Signaling Technology, Danvers, MA, USA, 4228S, 1:1000), PI3K (Cell Signaling Technology, Danvers, MA, USA, 4292S, 1:1000), p-AKT (Ser473) (Cell Signaling Technology, Danvers, MA, USA, 4060S, 1:1000), AKT (Cell Signaling Technology, Danvers, MA, USA, 4691S, 1:1000), p-CREB (Servicebio, Wuhan, China, GB114322, 1:1000), and CREB (Servicebio, Wuhan, China, GB111052, 1:1000), p-GSK3β (Ser9) (Cell Signaling Technology, Danvers, MA, USA, 9336S, 1:1000), GSK3β (Cell Signaling Technology, Danvers, MA, USA, 9315S, 1:1000), and p-Tau (Ser404) (Proteintech, Wuhan, China, 81383-1-RR, 1:5000). With GAPDH (Servicebio, Wuhan, China, GB15002, 1:5000) and β-Tubulin (Servicebio, Wuhan, China, GB122667, 1:5000) as loading controls.

### 2.13. Statistics

Statistical analyses were conducted utilizing GraphPad Prism version 10 (GraphPad Software, La Jolla, CA, USA). Data are expressed as mean ± SEM, with each hollow dot on the bar representing an individual sample measurement. Group comparisons were performed via one-way or two-way ANOVA, followed by Tukey’s post hoc test for multiple comparisons when appropriate. Statistical significance was established at *p* < 0.05, indicated by * symbols.

## 3. Results

### 3.1. The Beneficial Effect of MSA on Scopolamine-Induced Mice

#### 3.1.1. MsA Alleviates Cognitive Impairments in Mice Induced with Scopolamine

To examine the effects of MsA on recognition memory, the NOR test was conducted following the schematic design shown in Figure 1B. Mice treated with scopolamine showed a significant reduction in exploration time of the novel object at both 5 and 10 min, compared to the control group (Figure 1D,E), indicating impaired recognition memory. Treatment with MsA (30 mg/kg) significantly increased novel object exploration at both time points, effectively reversing scopolamine-induced deficits, while DNP (3 mg/kg) treatment led to a significant improvement only at 10 min, with no statistically significant change observed at 5 min. Furthermore, both MsA and DNP treatments significantly improved the discrimination index at 10 min compared to the SCOP group (Figure 1F), suggesting enhanced recognition memory performance.

In the MWM test, representative swim paths (Figure 1G) in the probe test demonstrated that MsA treatment led to a significant decrease in escape latency over five days of training (Figure 1H). Furthermore, MsA significantly increased crossing times of the platform (Figure 1I), time spent in the target quadrant (Figure 1J), and distance traveled in the target quadrant (Figure 1K), suggesting enhanced spatial memory and cognitive function in the MsA-treated group. These results indicate that MsA has a positive effect on cognitive function and memory retention in mice induced with scopolamine.

#### 3.1.2. MsA Reduces Neuronal Loss in the Hippocampus and Cortex in Mice Induced with Scopolamine

**Figure 2:**
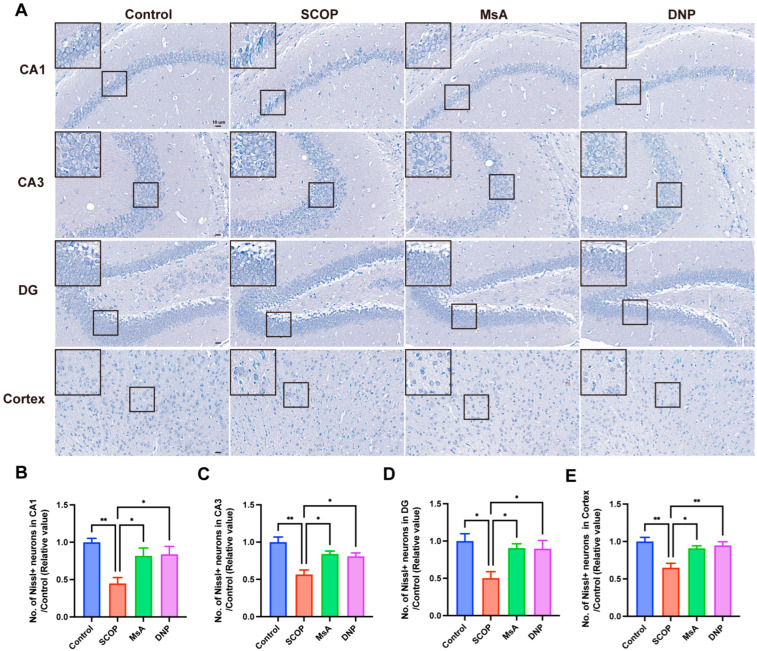
MsA reduces neuronal loss in the hippocampus and cortex in mice induced with scopolamine. (**A**) Representative Nissl-stained images of the CA1, CA3, DG, and cortex regions from the control, SCOP, MsA-treated, and DNP-treated groups. Insets highlight neuronal morphology and density. Scale bar measures 10 μm. (**B**–**E**) Quantification of Nissl-positive neurons in the CA1 (**B**), CA3 (**C**), DG (**D**), and cortex (**E**) regions. Data are expressed as mean ± SEM (*n* = 3). * *p* < 0.05, ** *p* < 0.01 vs. SCOP group.

To evaluate the neuroprotective effects of MsA on scopolamine-induced neuronal damage, Nissl staining was used to evaluate neuronal integrity in important brain areas, such as the hippocampal CA1, CA3, DG regions, and cortex. Representative images (Figure 2A) revealed that the SCOP group exhibited a marked reduction in Nissl-positive neurons across all examined areas compared to the control group, indicating significant neuronal loss. MsA treatment notably elevated the count of Nissl-positive neurons in the CA1, CA3, DG regions, and cortex regions relative to the SCOP group, suggesting an improvement in neuronal survival. Similarly, mice treated with donepezil demonstrated a significant increase in Nissl-positive neurons relative to the SCOP group. Quantitative analysis further confirmed these observations (Figure 2B–E). These findings indicate that MsA administration effectively alleviated scopolamine-induced neuronal loss in multiple brain regions associated with cognitive function.

#### 3.1.3. MsA Regulates Cholinergic Metabolism in the Hippocampus and Cortex of Scopolamine-Treated Mice

**Figure 3:**
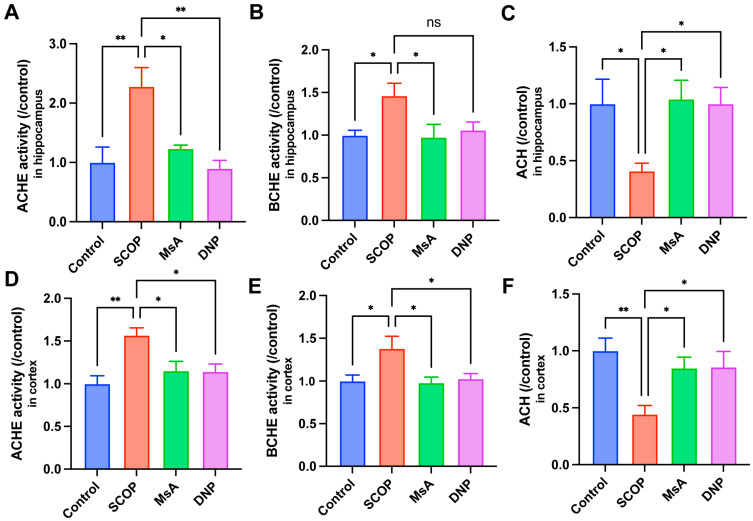
MsA regulates cholinergic metabolism in the hippocampus and cortex of scopolamine-treated mice. (**A**–**C**) AChE activity (**A**), BChE activity (**B**), and acetylcholine ACh levels (**C**), in the hippocampus. (**D**–**F**) AChE activity (**D**), BChE activity (**E**), and ACh levels (**F**) in the cortex. Data are presented as mean ± SEM (*n* = 4). * *p* < 0.05, ** *p* < 0.01 vs. SCOP group. ns, no significance.

Scopolamine, a competitive antagonist of muscarinic acetylcholine receptors, induces systemic cholinergic impairment through receptor blockade. To elucidate the neuromodulatory capacity of MsA on cholinergic homeostasis, we quantitatively assessed regional variations in acetylcholine (ACh) metabolism by analyzing both hydrolytic enzymes (AChE and BChE) and neurotransmitter dynamics in the hippocampus and cortex. As demonstrated in Figure 3A–C, SCOP administration significantly increased AChE and BChE activities while reducing ACh levels in the hippocampus compared to the control group, indicating cholinergic dysfunction. Treatment with MsA significantly attenuated SCOP-induced elevations in both AChE and BChE activities, and partially restored hippocampal ACh levels, suggesting a protective modulation of cholinergic neurotransmission. Consistent results were observed in the cortex (Figure 3D–F). SCOP-treated mice displayed elevated cortical AChE and BChE activities and significantly reduced ACh levels relative to the controls. MsA administration markedly suppressed the increase in AChE and BChE activities, and significantly reversed the decline in ACh levels, with effects comparable to those seen with DNP, the positive control.

#### 3.1.4. MsA Modulates BDNF and CREB Levels in the Hippocampus and Cortex of Mice Treated with Scopolamine

**Figure 4:**
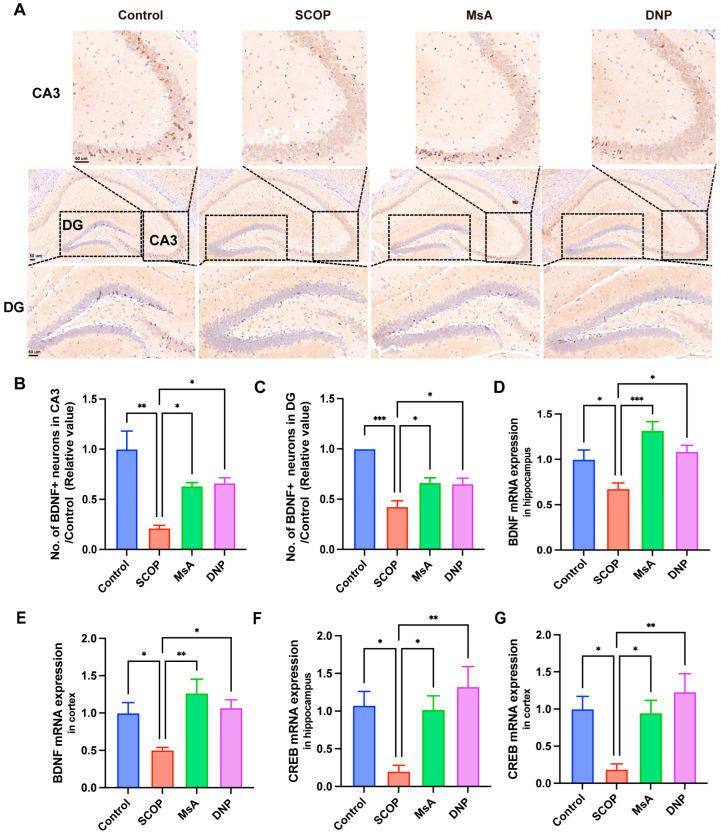
MsA regulates BDNF and CREB expression in the hippocampus and cortex of scopolamine-treated mice. (**A**) Representative immunohistochemical images showing BDNF-positive neurons in the CA3 and DG regions across different treatment groups. Insets highlight neuronal staining patterns. Scale bar measures 50 μm. (**B**,**C**) Quantification of BDNF-positive neurons in the CA3 (**B**) and DG (**C**) regions. (**D**,**E**) Relative *BDNF* mRNA expression levels in the hippocampus (**D**) and cortex (**E**). (**F**,**G**) Relative *CREB* mRNA expression levels in the hippocampus (**F**) and cortex (**G**). Data are presented as mean ± SEM (*n* = 3–5). * *p* < 0.05, ** *p* < 0.01, *** *p* < 0.001 vs. SCOP group.

As depicted in Figure 4A–C, immunohistochemical staining of the CA3 and DG regions revealed that scopolamine administration markedly decreased the count of BDNF-positive neurons relative to control mice. In contrast, MsA treatment notably elevated the count of BDNF-positive neurons in these hippocampal subregions compared to the SCOP group and comparable to the effects observed with DNP treatment. At the molecular level, qRT-PCR analysis demonstrated that *BDNF* mRNA expression in the hippocampus (Figure 4D) and cortex (Figure 4E) was significantly downregulated following scopolamine exposure. MsA administration effectively reversed these declines, significantly elevating *BDNF* mRNA levels compared to the SCOP group. Similarly, *CREB* mRNA expression, a key regulator of BDNF transcription, was markedly decreased in both the hippocampus (Figure 4F) and cortex (Figure 4G) following scopolamine treatment. MsA intervention significantly restored *CREB* mRNA expression in both brain regions.

### 3.2. The Impact of MSA on N2a/APP695swe Cells

#### 3.2.1. MsA Enhances Cholinergic System Function and Reduces Oxidative Damage in N2a/APP695swe Cells

**Figure 5:**
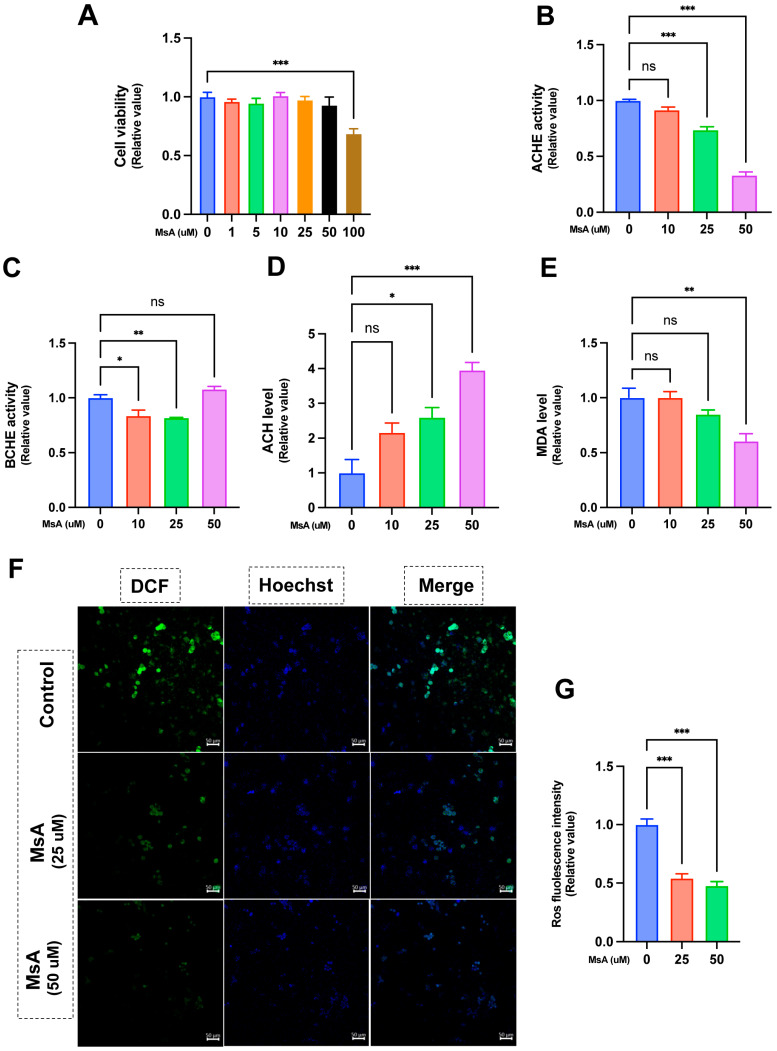
MsA enhances cholinergic system function and attenuates oxidative damage in N2a/APP695swe Cells. (**A**) The viability of N2a/APP695 cells subjected to 0–100 uM MsA for 24 h. (**B**–**D**) Effects of MsA on cholinergic system function, AChE activity (**B**), BChE activity (**C**), and ACh levels (**D**). (**E**–**G**) Effects of MsA on oxidative stress, MDA levels measured via TBARS assay (**E**). Representative fluorescence images of ROS detection (DCFH-DA, green), nuclear staining (Hoechst 33342, blue), and merged channels. Scale bar measures 50 μm (**F**), quantitative assessment of ROS fluorescence intensity (**G**). Data are presented as mean ± SEM (*n* = 3–6). * *p* < 0.05, ** *p* < 0.01, *** *p* < 0.001, compared with untreated N2a/APP695 cells. ns, no significance.

N2a/APP695swe cells underwent treatment with escalating concentrations of MsA (0, 1, 5, 10, 25, 50, and 100 μM) for a duration of 24 h. The viability of N2a/APP695swe cells was evaluated using the CCK-8 assay, revealing that MsA treatment did not significantly impact cell viability at concentrations up to 50 μM, with a reduction noted only at 100 μM (Figure 5A). MsA treatment notably enhanced AChE activity, with the most significant effects seen at 25 and 50 μM, while lower concentrations showed no significant changes (Figure 5B). Additionally, BChE activity was significantly reduced at 10 and 25 μM; however, no statistical reduction was observed at 50 μM (Figure 5C). In contrast, ACh levels increased at 25 and 50 μM, while no significant change was observed at 10 μM (Figure 5D).

To assess the impact of MsA on oxidative stress, MDA levels were measured using the TBARS assay. Treatment with 50 μM MsA caused a significant decrease in MDA levels compared to untreated cells, while no significant changes were detected at 10 and 25 μM (Figure 5E). Furthermore, ROS levels were measured by assessing DCFH-DA fluorescence, reflecting ROS accumulation. Representative images (Figure 5F) revealed that MsA treatment, particularly at 50 μM, significantly decreased ROS fluorescence intensity. Quantitative analysis of ROS intensity further verified a significant decrease in ROS levels, at both 25 and 50 μM concentrations of MsA compared to control cells (Figure 5G).

#### 3.2.2. MsA Modulates APP Processing-Related Gene and Protein Expression to Reduce Aβ1–42 Levels in N2a/APP695swe Cells

**Figure 6:**
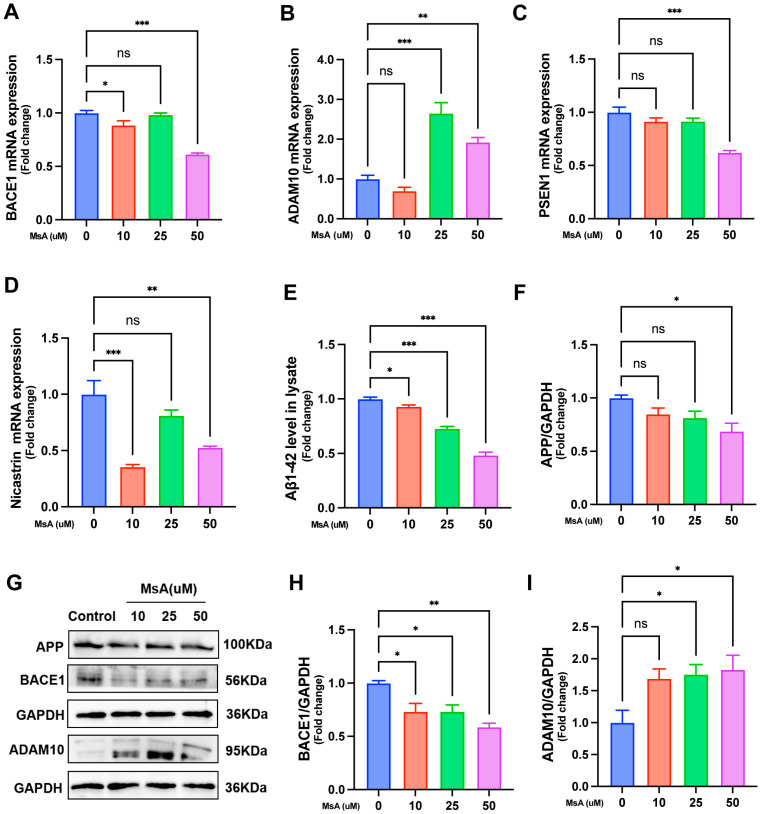
MsA modulates the expression of APP processing-related genes and proteins to reduce Aβ1–42 levels in N2a/APP695swe cells. (**A**–**D**) mRNA expression levels of *ADAM10* (**A**), *BACE1* (**B**), *PSEN1* (**C**), and *Nicastrin* (**D**) were determined using qRT-PCR. (**E**) Levels of Aβ1–42 in cell lysates were analyzed. Representative Western blot images (Supplementary Material Section S2.1) and (**F**–**I**) quantification of APP, BACE1, and ADAM10 protein levels are presented. GAPDH served as a loading control. Data are presented as mean ± SEM (*n* = 3) (Supplementary Material Section S2.2). * *p* < 0.05, ** *p* < 0.01, *** *p* < 0.001 in comparison to untreated control cells. ns, no significance.

The impact of MsA on the mRNA expression of genes involved in APP processing was examined using qRT-PCR. The results showed that MsA treatment significantly decreased *BACE1* expression at both 10 and 50 μM, with no change observed at 25 μM (Figure 6A). In contrast, *ADAM10* expression significantly increased at 25 and 50 μM, while no changes were observed at 10 μM (Figure 6B). For *PSEN1* (Figure 6C), a significant decrease in mRNA expression was detected at 50 μM, whereas no significant differences were observed at 10 or 25 μM. *Nicastrin* mRNA expression (Figure 6D) was significantly reduced at both 10 and 50 μM, with no significant change at 25 μM.

In terms of protein expression, ELISA analysis revealed that Aβ1–42 levels in N2a/APP695swe cell lysates were significantly reduced at 10, 25, and 50 μM MsA treatment (Figure 6E). Western blot analysis (Figure 6G) showed that MsA treatment altered protein levels of APP, BACE1, and ADAM10, with no changes in GAPDH loading control. Protein quantification indicated a significant decrease in APP at 50 μM and BACE1 at 10, 25, and 50 μM, while ADAM10 increased at 25 and 50 μM concentrations of MsA (Figure 6F,H,I).

#### 3.2.3. MsA Regulates the PI3K/AKT/GSK3β/CREB Signaling Pathway and Reduces Tau Phosphorylation in N2a/APP695swe Cells

**Figure 7:**
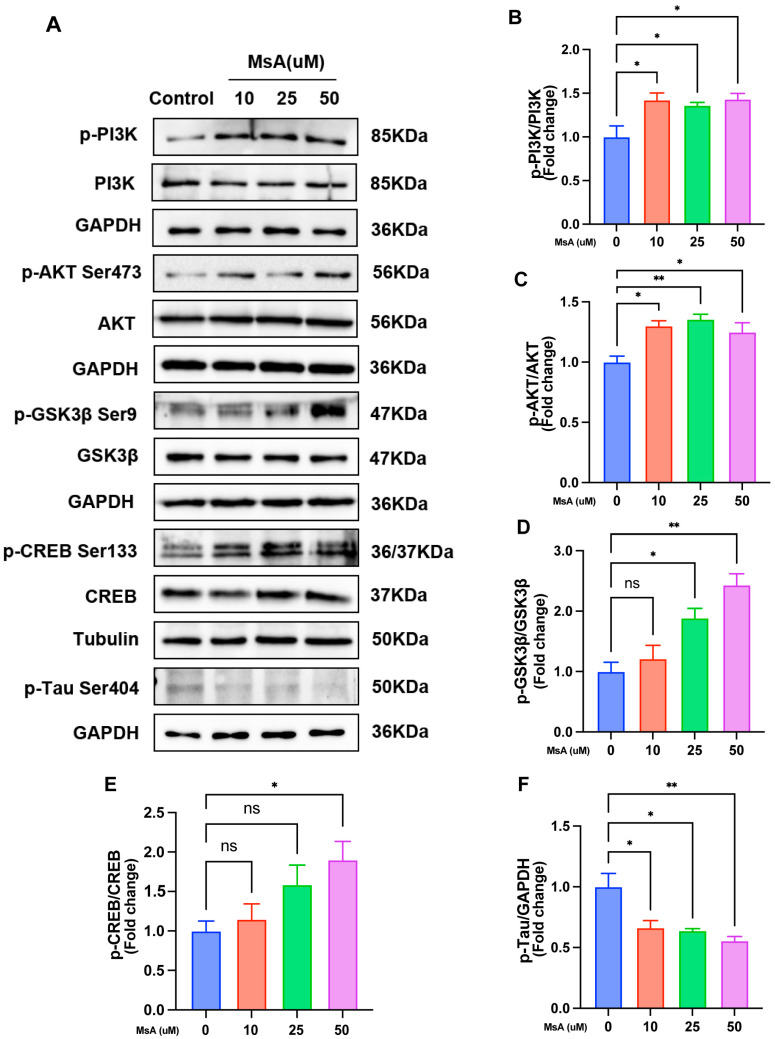
MsA regulates the PI3K/AKT/GSK3β/CREB signaling pathway and reduces tau phosphorylation in N2a/APP695swe cells. (**A**) Representative Western blot images (Supplementary Material Section S2.1) displaying the expression levels of phosphorylated PI3K (p-PI3K), total PI3K, phosphorylated AKT (p-AKT Ser473), total AKT, phosphorylated CREB (p-CREB), total CREB, phosphorylated GSK3β (p-GSK3β Ser9), total GSK3β, and phosphorylated Tau (p- Tau Ser404) in N2a/APP695swe cells treated with different concentrations of MsA (10, 25, 50 μM). GAPDH and tubulin served as loading controls. (**B**–**F**) Quantification of protein phosphorylation levels of p-PI3K/PI3K (**B**), p-AKT/AKT (**C**), p-CREB/CREB (**D**), p-GSK3β/GSK3β (**E**), and p-Tau/GAPDH (**F**). Data are presented as mean ± SEM (*n* = 3) (Supplementary Material Section S2.2). * *p* < 0.05, ** *p* < 0.01 vs. control group. ns, no significance.

To understand the molecular mechanisms by which MsA ameliorates Alzheimer’s-related cognitive deficits, we investigated its regulatory effects on the PI3K/AKT signaling axis in N2a/APP695swe cells. Western blotting analysis showed that MsA treatment (10–50 μM) significantly increased phosphorylation of key components, including PI3K, AKT (Ser473), and CREB, while promoting inhibitory phosphorylation of GSK3β (Ser9). Furthermore, MsA treatment (10–50 μM) significantly decreased the phosphorylation of tau at the Ser404 site (p-Tau Ser404), a key pathological feature in AD (Figure 7A). Quantitative analysis of phosphorylated-to-total protein ratios indicated activation of the PI3K/AKT/GSK3β/CREB cascade and the reduction in tau phosphorylation, with normalization to GAPDH and tubulin confirming the stable expression of pathway components (Figure 7B–F).

**Figure 8:**
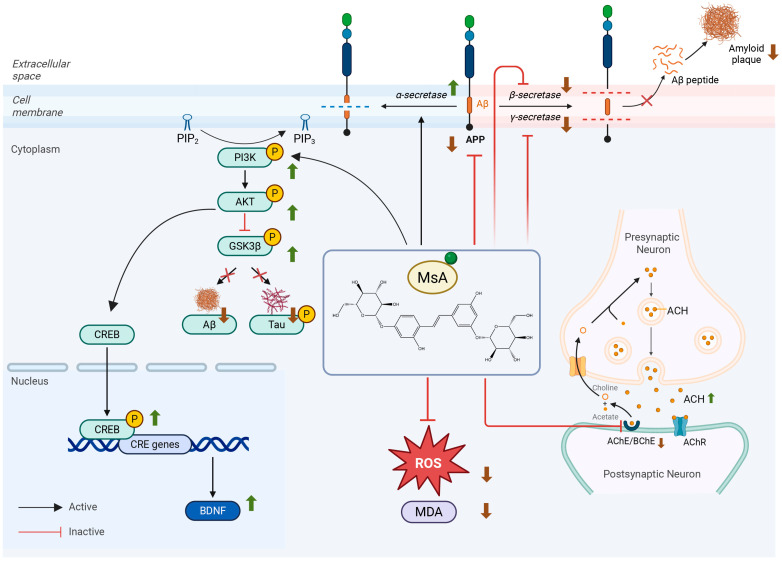
Mechanism diagram illustrating the neuroprotective effects of MsA in AD. MsA exerts its therapeutic effects through (1) Ameliorating cholinergic dysfunction by inhibiting AChE and BChE, increasing synaptic ACh; (2) Reducing oxidative stress markers (ROS, MDA); (3) Regulating APP processing via upregulating ADAM10 (non-amyloidogenic pathway) while suppressing BACE1 and γ-secretase (amyloidogenic pathway), thereby decreasing Aβ production; (4) Activating the PI3K/AKT pathway, leading to inhibitory phosphorylation of GSK3β (reducing tau hyperphosphorylation) and activation of CREB/BDNF signaling. Created with BioRender. Tu, D. (2025) https://BioRender.com/be7iclu.

Collectively, the schematic diagram (Figure 8) integrates our findings to illustrate the multi-target neuroprotective mechanism of MsA against Alzheimer’s disease pathology. MsA ameliorates cholinergic dysfunction by inhibiting AChE and BChE activities, thereby increasing synaptic Ach levels and enhancing neurotransmission. Furthermore, MsA mitigates oxidative stress by reducing markers like ROS and MDA. Crucially, MsA targets amyloid-β (Aβ) pathogenesis through dual regulation of APP processing: it promotes the non-amyloidogenic pathway by upregulating ADAM10 (α-secretase) and concurrently inhibits the amyloidogenic pathway by suppressing BACE1 (β-secretase) and γ-secretase components, ultimately reducing Aβ production. Mechanistically, these diverse benefits converge on MsA’s activation of the PI3K/AKT signaling pathway. This activation triggers the inhibitory phosphorylation of GSK3β, which directly reduces pathogenic tau hyperphosphorylation. Simultaneously, PI3K/AKT activation stimulates the CREB, leading to upregulated BDNF expression.

## 4. Discussion

AD is a neurological condition primarily impacting the elderly, resulting in considerable health impairments. However, effective therapeutic options remain limited. Recent studies indicate that *Morus alba* L. root cortex extract can improve cognitive function in AD-like animal models by modulating key AD-related factors such as AChE, BChE, BACE1, APP, tau, and Aβ [22,23,24]. MsA, a major bioactive component of *Morus alba* L., has demonstrated substantial neuroprotective effects [21,32,33]. The 30 mg/kg dose was chosen based on previous studies involving doses of 5–50 mg/kg that did not report significant toxicity (Supplementary Material Section S1.3) [19,33,34,35,36,37,38,39]. Particularly in a study on MsA’s neuroprotective effects in mice fed a high-fructose diet, MsA (20 and 40 mg/kg) reduced hippocampal neuroinflammation and impairment neurogenesis [33]. Additionally, our safety assessments also followed body weight weekly during treatment and brain, kidney, and liver organ weights when sacrificed, finding no significant variations between groups (Supplementary Material Section S1.2). Based on this overall evidence, we assessed 30 mg/kg of MsA’s therapeutic potential in scopolamine-induced cognitive impairment in mice. The novel object recognition behavioral test revealed that MsA significantly increased novel object exploration in the 5 and 10 min tests. Moreover, in the water maze tests, MsA treatment led to a notable reduction in escape latency during five days of training and significantly increased the crossing times of the platform, time spent in target quadrant, and distance traveled in target quadrant (Figure 1 and Supplementary S1). These results indicate that MsA could help prevent or manage AD-related symptoms, reinforcing its potential as a promising therapy for cognitive decline in AD.

Our research on MsA in N2a/APP695swe cells and scopolamine-induced cognitive impairment mouse models demonstrates that MsA significantly elevates ACh levels while effectively inhibiting AChE and BChE enzymes, highlighting its regulatory function in the cholinergic system. Recent studies have also highlighted that suppressing BChE is pivotal in the etiology of AD, particularly when AChE levels decrease, as the compensatory overexpression of BChE may worsen cholinergic dysfunction [40,41]. Our experimental findings support this idea, demonstrating that MsA, by reducing BChE activity, not only decreases ACh hydrolysis but may also hinder BChE’s involvement in amyloid beta (Aβ) deposition (Figure 3, Figure 5B,D and Figure 6E). Prior research indicates that BChE accumulation is closely linked to Aβ plaque production. In BChE knockout animal models, Aβ deposition is significantly reduced, and cognitive function improves, reinforcing the potential of BChE as a promising therapeutic target AD [42,43]. Consequently, MsA may effectively alleviate cholinergic insufficiency and neurotoxicity associated with Aβ accumulation in AD through the dual suppression of AChE and BChE activity, offering novel insights and methods for treatment.

We further investigated the impact of MsA on APP metabolism in N2a/APP695swe cells, a recognized cellular model that overexpresses the Swedish-mutated APP, and found that MsA significantly reduced Aβ1–42 levels. It is well-established that preventing Aβ aggregation and inhibiting Aβ generation are crucial for managing and potentially preventing AD. The processing of APP results in the generation of Aβ peptides, and the equilibrium between amyloidogenic and non-amyloidogenic pathways is crucial in AD progression [44]. In particular, modulating the activity of APP-cleaving enzymes—namely α-, β-, and γ-secretases—has proven to effectively decrease Aβ production and deposition in the AD brain [8,45]. In our study, MsA was found to downregulate the expression of BACE1 and γ-secretase components, including PSEN1 and Nicastrin, which participate in the amyloidogenic processing of APP. Concurrently, MsA upregulated ADAM10, an α-secretase linked to the non-amyloidogenic pathway (Figure 6), suggesting that MsA may shift APP processing toward a less harmful pathway, thereby reducing the production of neurotoxic Aβ species.

Oxidative stress possesses neurotoxic potential and is instrumental in neurodegeneration [46,47]. The progression of AD is characterized by the abnormal accumulation of Aβ aggregates, which arises from elevated free radicals or impaired antioxidant mechanisms, leading to oxidative stress and neurotoxicity in the brain [48]. Our findings align with this idea, as we showed that MsA, particularly at 50 μM, dramatically reduced levels of MDA and ROS in the N2a/APP695swe cell model (Figure 5E–G). By reducing MDA and ROS levels, MsA appears to alleviate the oxidative stress associated with Aβ buildup, thereby protecting neurons from the harmful effects of oxidative damage. These data further suggest that MsA could reduce oxidative stress in the brain, a key factor in the progression of AD. Additionally, in the scopolamine-induced cognitive impairment animal model, MsA therapy led to significant increases in neuronal integrity in both the hippocampus and cortex, as demonstrated by Nissl staining (Figure 2). These findings highlight the potential of MsA to not only reduce oxidative stress but also enhance neuronal health and cognitive performance.

The PI3K/AKT pathway is a recognized signaling system associated with insulin resistance, believed to be modified throughout the progression of AD [49]. As a central node linking Aβ accumulation and tau hyperphosphorylation, GSK3β directly phosphorylates tau at over 20 serine/threonine, while concurrently promoting Aβ generation via BACE1/γ-secretase activation [50]. GSK3β activation is essential for the formation and accumulation of Aβ in the brains of AD, as it influences the cleavage of APP [50]. We investigated the phosphorylation of key signaling molecules—PI3K (Tyr467), AKT (Ser473), GSK3β (Ser9), and Tau (Ser404)—in the N2a/APP695swe cellular AD model following MsA treatment. Our results indicate that MsA activates the PI3K/AKT/GSK3β pathway, as evidenced by the elevated phosphorylation levels of these components. We also found that MsA treatment (10–50 μM) significantly reduces tau phosphorylation at Ser404, mechanistically linking PI3K/AKT/GSK3β activation to tauopathy mitigation (Figure 7). The noted enhancement of PI3K-AKT signaling due to MsA, which leads to heightened inhibitory phosphorylation of GSK3β, is consistent with earlier studies indicating that this pathway could have positive effects on AD pathology [51].

Furthermore, phosphorylated (inactive) GSK3β translocates to the nucleus where it can interact with transcription factors, including cAMP response element-binding protein (CREB) [52]. CREB is essential for the survival of neurons, memory consolidation, and synaptic plasticity, and its dysfunction is closely associated with cognitive impairment in AD. Brain-derived neurotrophic factor (BDNF) is a critical downstream target of CREB, as it is vital for the survival and function of neurons The cognitive decline in AD is strongly correlated with dysregulation of the CREB-BDNF signaling axis [53]. Our research indicates that the inhibition of GSK3β (via Ser9 phosphorylation) induced by MsA holds dual significance for CREB activation. Previous studies reveal that GSK3β directly phosphorylates CREB at Ser129, leading to its deactivation and a decrease in neuroprotective gene transcription [54,55]. Consequently, the MsA-induced inhibition of GSK3β likely alleviates this suppressive barrier on CREB, allowing for its phosphorylation at the activating Ser133 site. This molecular relationship is further supported by our observation of increased phosphorylated CREB levels in MsA-treated mice (Figure 4F,G and Figure 7A,D). Additionally, pCREB serves as a key transcriptional regulator of BDNF, a neurotrophin whose deficiency is mechanistically linked to Aβ toxicity and synaptic dysfunction in AD [56,57,58]. Aβ oligomers significantly reduce BDNF expression, partly due to CREB dysfunction [56,57], while the restoration of CREB/BDNF signaling improves cognitive deficits, regardless of Aβ pathology, in various models [59,60]. The findings showing an MsA-induced increase in BDNF mRNA and protein expression in the hippocampus reinforce this established relationship (Figure 4A–E), suggesting that MsA may help counteract Aβ-induced BDNF depletion through the PI3K/AKT/GSK3β-mediated activation of CREB.

The multi-target neuroprotective profile of MsA observed in this study reveals both similarities and unique characteristics compared to established neuroprotective compounds like curcumin, resveratrol, and ginsenosides. While these compounds share antioxidant and Aβ-modulating capabilities [61,62,63], MsA advances cholinergic intervention through multiple levels of experimental validation. Unlike most natural product studies that often focus on in silico docking or only in vitro evaluations of cholinesterase inhibition, our research provides substantial in vivo and in vitro evidence that MsA significantly increases Ach levels while inhibiting both AChE and BChE enzymes (Figure 3 and Figure 5B–D). MsA’s suppression of BChE, an enzyme overexpressed in late-stage AD and contributing to Aβ deposition and cognitive decline, represents a strategically important therapeutic intervention that has not been thoroughly documented for comparator compounds. Furthermore, although PI3K/AKT activation is common to both curcumin [64] and ginsenosides [65], MsA interacts with a broader signaling axis. Our data indicates that MsA activates the entire PI3K/AKT/GSK3β/CREB/BDNF cascade, initiating downstream neuroplasticity and survival signals in vivo. This report provides initial evidence of MsA’s neuroprotective effects in AD models, emphasizing its distinct mechanisms in addressing the complex nature of AD pathogenesis.

This study has several limitations. First, while the scopolamine model effectively induces cholinergic dysfunction—a core feature of AD—and replicates early cognitive deficits, it does not fully recapitulate the progressive neurodegeneration or complex proteinopathy characteristic of clinical AD. Specifically, chronic scopolamine exposure does not produce mature Aβ plaques or neurofibrillary tangles and lacks region-specific pathological progression, despite reported transient elevations in soluble Aβ and phospho-tau. Consequently, therapeutic effects observed in this model may not fully translate to advanced AD pathology. Second, although oxidative stress represents a mechanistically relevant AD pathway detected in vitro, we did not assess this endpoint in scopolamine-treated mice, limiting our understanding of MsA’s in vivo antioxidant potential. Third, modest cohort sizes in histological analyses may constrain statistical power despite significant findings—future studies would benefit from expanded sampling. Fourth, while no acute toxicity was observed, the absence of chronic safety assessments necessitates further long-term evaluation. Finally, although we focused on PI3K/AKT/GSK3β/CREB signaling, other AD-relevant mechanisms remain unexplored. Recent advances in computational modeling, particularly in simulating cognitive processes associated with AD, have provided valuable insights [66]. Future studies could leverage mathematical models to further deepen our understanding of the overall therapeutic potential of MsA in AD, offering a complementary approach to experimental research.

## 5. Conclusions

This study explores the neuroprotective effects of MsA on AD. We systematically examined how MsA influences key aspects of AD pathology, including cholinergic dysfunction, amyloid-β buildup, tau hyperphosphorylation and oxidative stress, using both in vitro and in vivo models. In the in vivo scopolamine-induced model, MsA showed a significant improvement in cognitive function and a decrease in cholinergic deficits, as evidenced by behavioral testing, Nissl staining, and biochemical analyses. In vitro, MsA demonstrated the ability to enhance cholinergic function, reduce oxidative stress, suppressed pathological tau modifications, and decrease Aβ production by modulating amyloidogenic processing. Mechanistic studies indicated that MsA activated the PI3K/AKT pathway, which inhibited GSK3β activity and promoted CREB phosphorylation, leading to reduced tau phosphorylation and increased BDNF levels. The findings suggest that MsA provides multi-target neuroprotection against AD’s key pathologies: amyloid plaques, neurofibrillary tangles, cholinergic degeneration, and oxidative stress, via the PI3K/AKT/GSK3β/CREB signaling pathway. This indicates that MsA is a promising therapeutic candidate for AD.

## Figures and Tables

**Table 1:** Nucleotide sequence of qPCR primer.

Target	Primer Sequence (5′ to 3′)	Primer Length (bp)
*BACE1*	F: CTGCCATCACTGAATCGGACAAG R: TCACCAGGGAGTCAAGAAGGG	126
*ADAM10*	F: TTGATGATGGTGTTC R: GATGCCTGTGTTCAATCACTTCTTC	119
*PSEN1*	F: TGTGGTTGGTGAATATGGCTGAAG R: TCTCCGCTCTTTGTGTGTATACTTG	87
*Nicastrin*	F: TCTGCTCTATGGGTTCCTGGTTAG R: GAGACCGCCATGTAGTGTGAAG	114
*BDNF*	F: CGACGACATCACTGGCTGACAC R: GAGGCTCCAAAGGCACTTGACTG	150
*CREB*	F: CTGAAGAAGCAGCACGGAAGAGAG R: TTCAAGCACTGCCACTCTGTTCTC	122
*GAPDH*	F: AACTCCCACTCCTTCCACCTTCCG R: TCCACCACCCTGTTGCCTGTAG	113

Note: F (Forward), R (Reverse).

## Data Availability

Not applicable.

## Supplementary Materials

The following supporting information can be downloaded at: https://www.mdpi.com/article/10.3390/biology14091114/s1, S1.1: MWM test, representative swim paths of mice on the probe day; S1.2: Effects of MsA on body weight, brain, kidney and liver weights in scopolamine-treated mice; S1.3: Dosage ranges and toxicity observations of MsA in prior in vivo studies; Section S2.1 Raw membranes of Western blot with crop points; Section S2.2 Membranes employed in Western blot analysis.

## Abbreviations

The abbreviations used in this manuscript are as follows:

AD	Alzheimer’s disease
MsA	Mulberroside A
ACh	Acetylcholine
AChE	Acetylcholinesterase
BChE	Butyrylcholinesterase
Aβ	Amyloid-beta
BDNF	Brain-derived neurotrophic factor
CREB	cAMP response element-binding protein
APP	Amyloid precursor protein
SCOP	Scopolamine
DNP	Donepezil
NOR	Novel object recognition
MWW	Morris Water Maze
MDA	Malondialdehyde
CCK-8	Cell Counting Kit-8
ROS	Reactive oxygen species
qRT-PCR	Quantitative real-time PCR
