# Supplementary material for: Mulberroside A: A Multi-Target Neuroprotective Agent in Alzheimer’s Disease via Cholinergic Restoration and PI3K/AKT Pathway Activation
Source: Biology (Basel). 2025 Aug 22;14(9):1114. doi: 10.3390/biology14091114 (PMC12467343; doi:10.3390/biology14091114)
Supplement: Supplementary file 1 [file biology-14-01114-s001.zip › biology-3780640-supplementary.pdf]

Supplementary Materials

Index

Content

	Page
**Section S1. The Effect of MsA on Scopolamine-Induced Mice**	2
**Section S1.1** MWM test, representative swim paths of mice on the probe day.	2
**Section S1.2** Effects of MsA on body weight, brain, liver and kidney weights in scopolamine-treated mice.	3
**Section S1.3** Dosage ranges and toxicity observations of MsA in prior in vivo studies.	4
**Section S2. The Effect of MsA on N2a/APP695 Cells**	6
**Section S2.1** Raw membranes of Western blot with crop points	6
**Section S2.2** Membranes employed in Western blot analysis	12

Section S1. The Effect of MsA on Scopolamine-Induced Mice

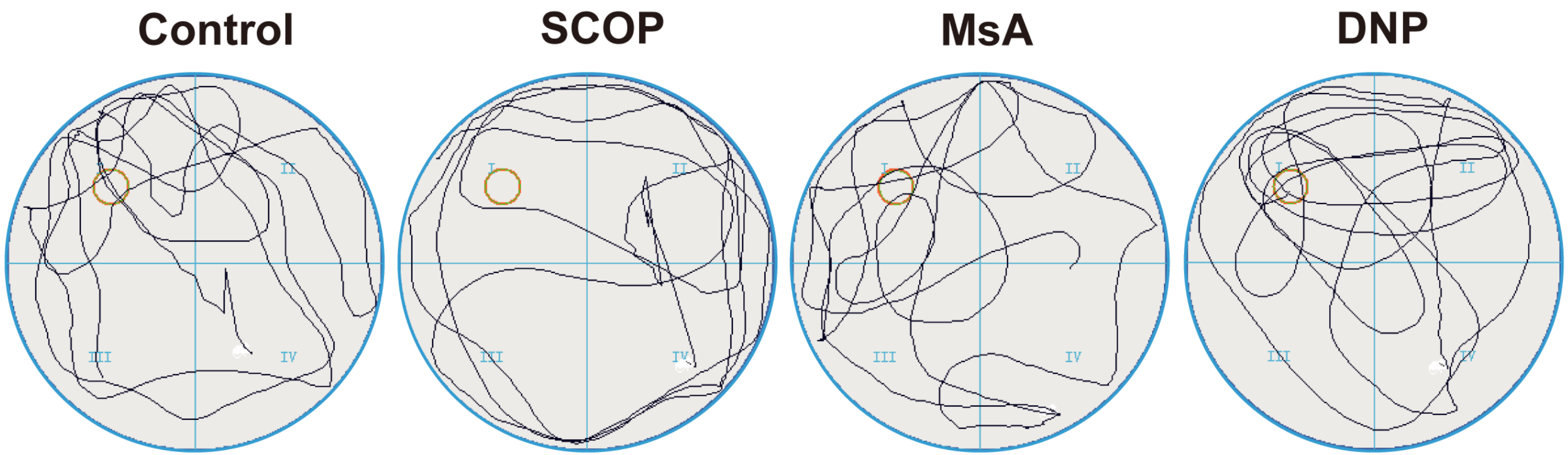

Section S1.1 MWM test, representative swim paths of mice on the probe day.

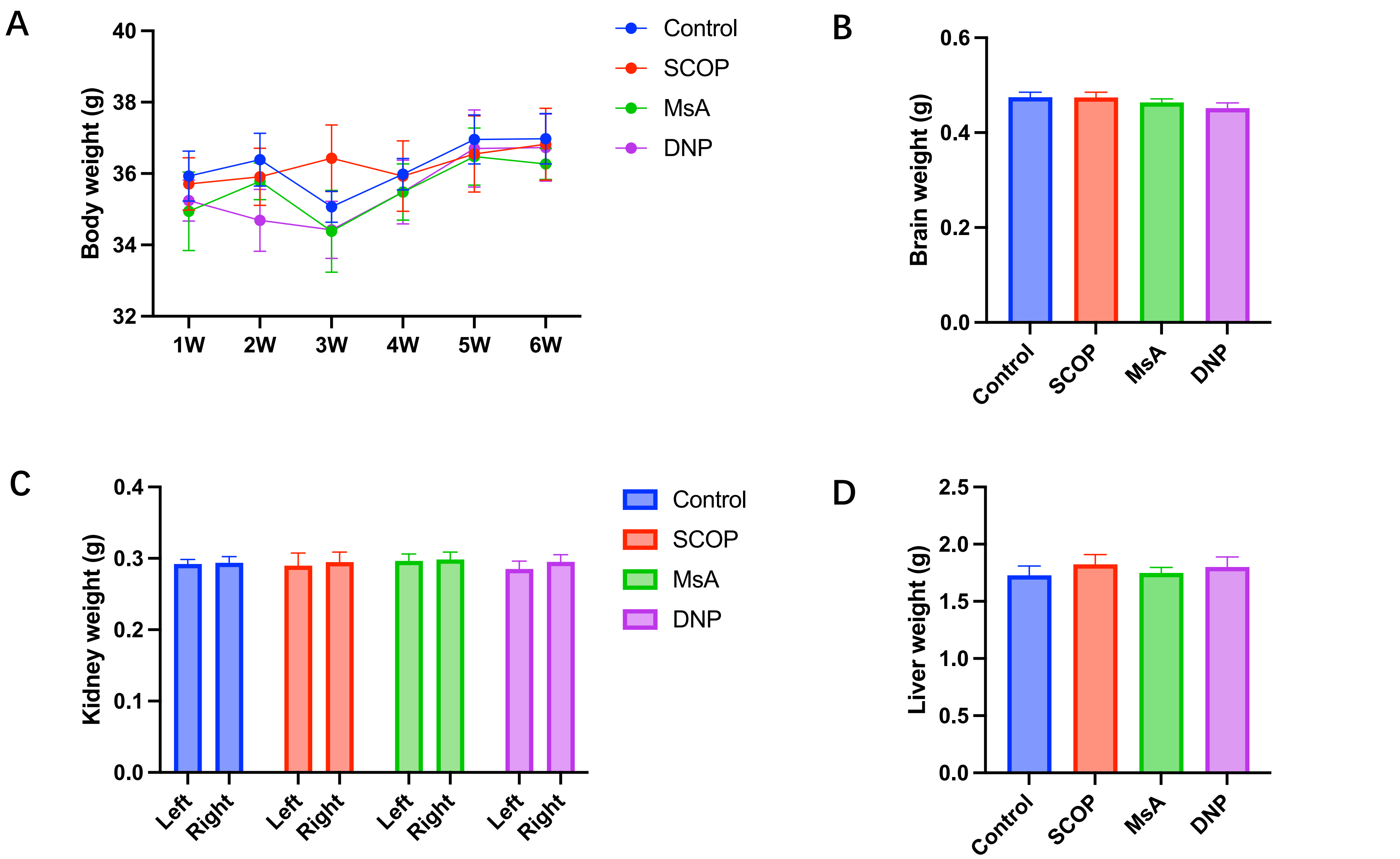

Section S1.2 Effects of MsA on body weight (A) , brain (B) , kidney (C) and liver (D) weights in scopolamine-treated mice.

Table. Dosage ranges and toxicity observations of MsA in prior in vivo studies.

Diseases	In- vivo models	Dosage and administration	Treatment time	Toxicity	References
AD	scopolamine-treated mice	30 mg/kg, oral	34 days	Not observed	Our study
Atherosclerosis	Cigarette smoke-induced ApoE− /− mice	40 mg/kg, not mentioned	12 weeks	Not mentioned	Liu et al., 2025
Osteoporosis	Ovariectomy in mice	20 , 40 mg/kg; i.p.	6 weeks	Not mentioned	Xue et al., 2024
Hepatic injury	CCl4 -induced mice; ethanol-induced mice	20 , 40 mg/g; oral; 30 , 60 mg/kg; oral	4 weeks, 7 days	Not mentioned	Shi et al., 2023; Zhang et al., 2008
Gut dysbiosis	High fructose diet-fed mice	20 , 40 mg/kg; oral	8 weeks	Not mentioned	Yu et al., 2021
Healthy	Rats	10, 20 , 40 mg/kg; oral	12h	Not mentioned	Li et al., 2014
Hyperuricemia	Oxonate-induced hyperuricemic mice	5, 10, 20 , 40 mg/kg; oral	7 days	Not mentioned	Wang et al., 2011
Inflflammation	Acetic acid-induced mice	25 , 50 mg/kg; oral	30min	Not mentioned	Zhang et al., 2010

Section S1.3 Dosage ranges and toxicity observations of MsA in prior in vivo studies.

References

1. Liu, T.; Zhang, Q.; Zhao, F.; Yin, J.; Liu, H.; Wang, L.; Liu, B. Mulberroside A Alleviates Myocardial Infarction by Inhibiting Oxidative Stress and Apoptosis via the PI3K/Akt Signaling Pathway. *Bratisl. Med. J.* **2025,** *2025, 1-9*. https://doi.org/10.1007/s44411-025-00197-3.
2. Xue, H.; Feng, Z.; Yuan, P.; Qiao, L.; Lou, Q.; Zhao, X.; Ma, Q.; Wang, S.; Shen, Y.; Ye, H.; et al. Restrained Mitf-Associated Autophagy by Mulberroside A Ameliorates Osteoclastogenesis and Counteracts OVX-Induced Osteoporosis in Mice. *Cell Death Discov.* **2024**, *10*, 80. https://doi.org/10.1038/s41420-024-01847-1.
3. Shi, B.; Qian, J.; Miao, H.; Zhang, S.; Hu, Y.; Liu, P.; Xu, L. Mulberroside A Ameliorates CCl4-Induced Liver Fibrosis in Mice via Inhibiting pro-Inflammatory Response. *Food Sci. Nutr.* **2023**, *11*, 3433–3441. https://doi.org/10.1002/fsn3.3333.
4. Zhang, Z.; Jin, J.; Shi, L. Protective Function of *Cis*-Mulberroside a and Oxyresveratrol from *Ramulus mori* against Ethanol-Induced Hepatic Damage. *Environ. Toxicol. Pharmacol.* **2008**, *26*, 325–330. https://doi.org/10.1016/j.etap.2008.06.008.
5. Yu, R.; Wen, S.; Wang, Q.; Wang, C.; Zhang, L.; Wu, X.; Li, J.; Kong, L. Mulberroside a Repairs High Fructose Diet-Induced Damage of Intestinal Epithelial and Blood-Brain Barriers in Mice: A Potential for Preventing Hippocampal Neuroinflammatory Injury. *J. Neurochem.* **2021**, *157*, 1979–1991. https://doi.org/10.1111/jnc.15242.
6. Li, Y.; Huang, L.; Zeng, X.; Zhong, G.; Ying, M.; Huang, M.; Bi, H. Down-Regulation of P-Gp Expression and Function after Mulberroside A Treatment: Potential Role of Protein Kinase C and NF-Kappa B. *Chem. Biol. Interact.* **2014**, *213*, 44–50. https://doi.org/10.1016/j.cbi.2014.02.004.
7. Wang, C.-P.; Wang, Y.; Wang, X.; Zhang, X.; Ye, J.-F.; Hu, L.-S.; Kong, L.-D. Mulberroside A Possesses Potent Uricosuric and Nephroprotective Effects in Hyperuricemic Mice. *Planta Med.* **2011**, *77*, 786–794. https://doi.org/10.1055/s-0030-1250599.
8. Zhang, Z.; Shi, L. Anti-Inflammatory and Analgesic Properties of *Cis*-Mulberroside A from *Ramulus mori*. *Fitoterapia* **2010**, *81*, 214–218. https://doi.org/10.1016/j.fitote.2009.09.005.

Section S2. The Effect of MsA on N2a/APP695 Cells

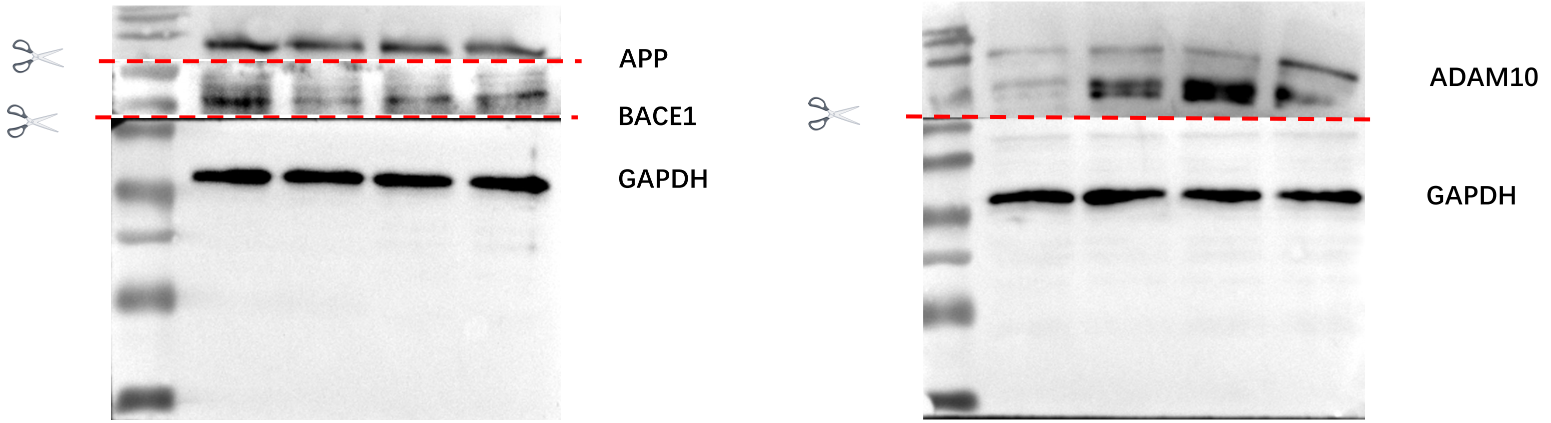

Figure 6G

Section S2.1 Raw membranes of Western blot with crop points

Section S2. The Effect of MsA on N2a/APP695 Cells

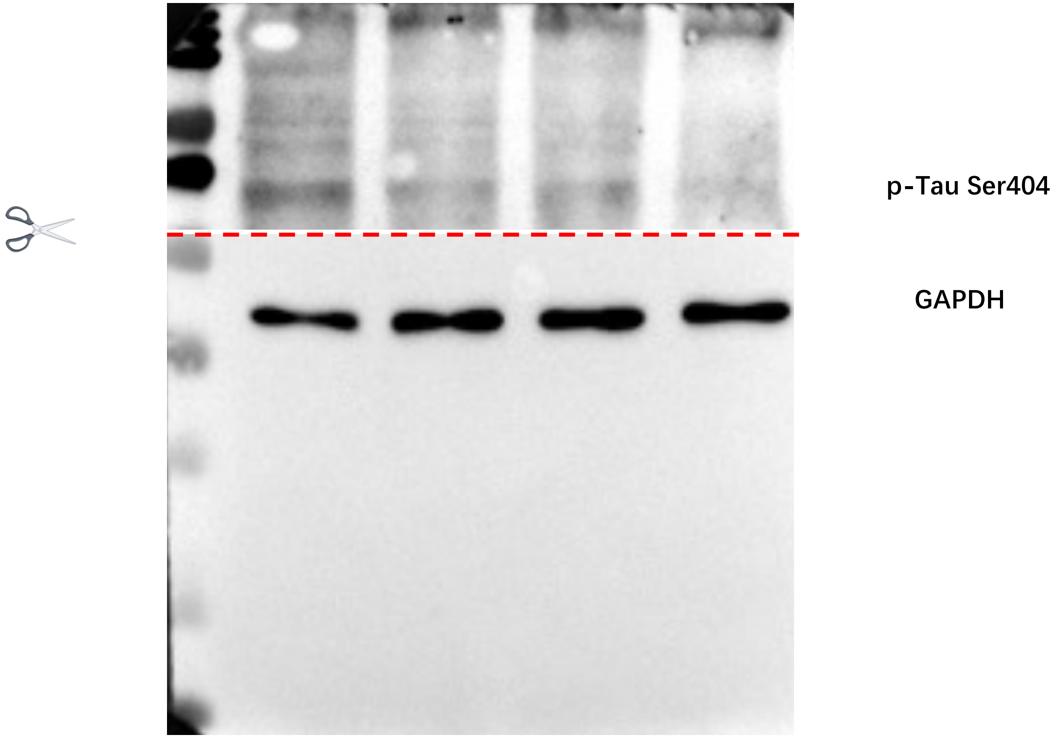

Figure 7A-1

Section S2.1 Raw membranes of Western blot with crop points

Section S2. The Effect of MsA on N2a/APP695 Cells

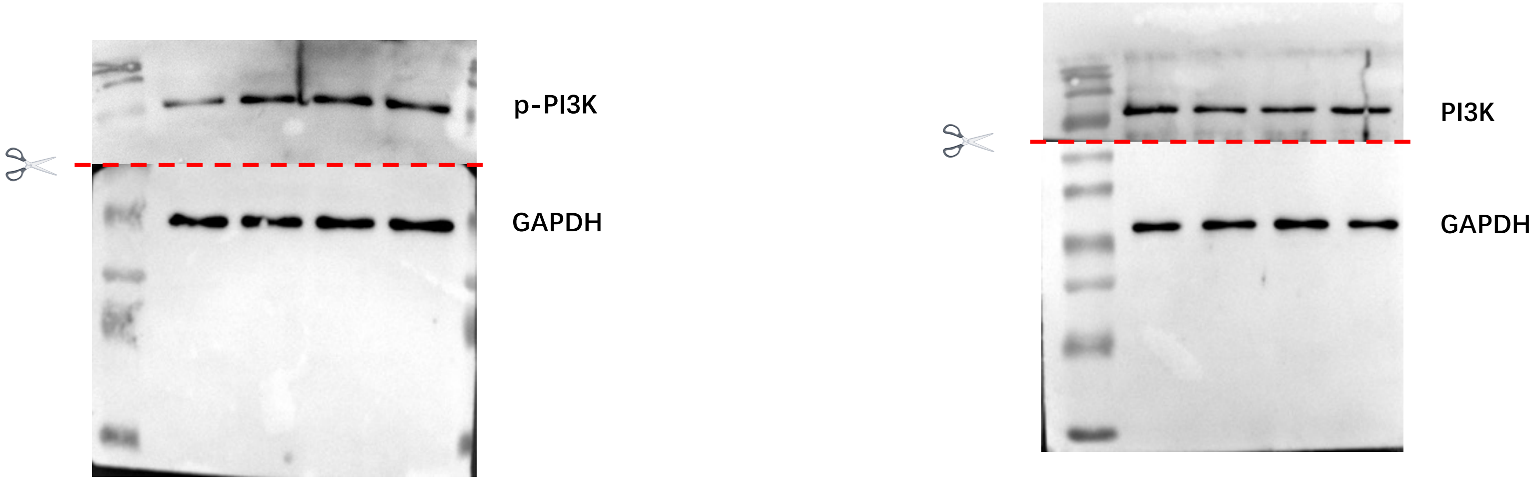

Figure 7A-2

Section S2.1 Raw membranes of Western blot with crop points

Section S2. The Effect of MsA on N2a/APP695 Cells

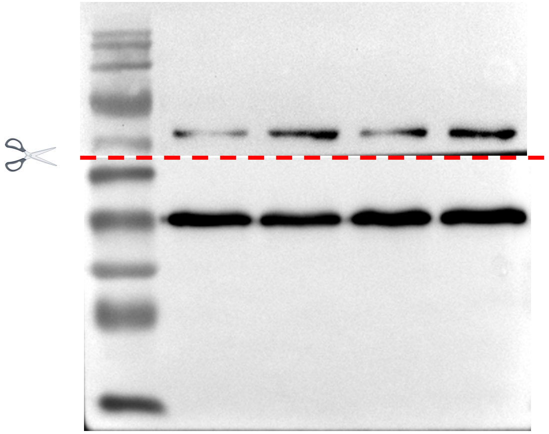

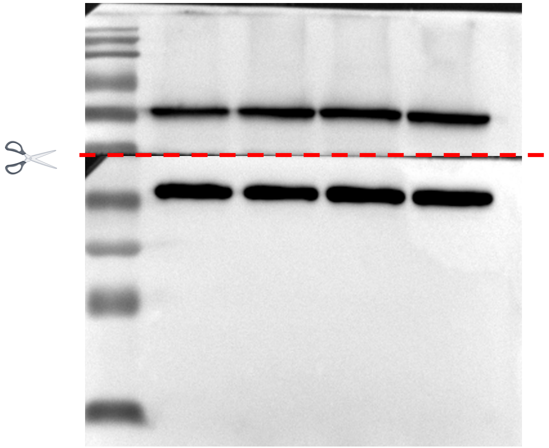

Figure 7A-3

Section S2.1 Raw membranes of Western blot with crop points

Section S2. The Effect of MsA on N2a/APP695 Cells

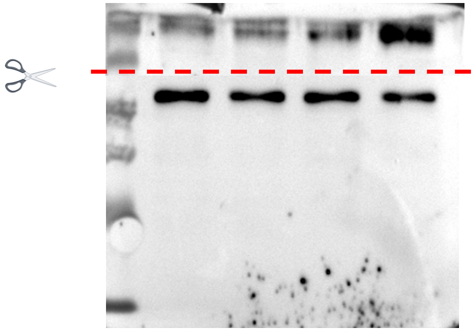

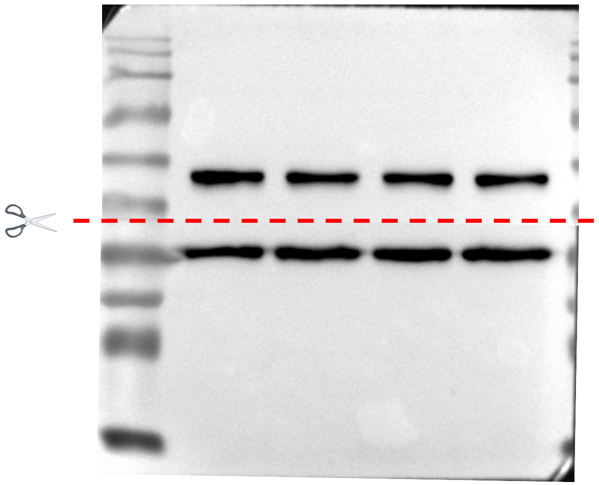

Figure 7A-4

Section S2.1 Raw membranes of Western blot with crop points

Section S2. The Effect of MsA on N2a/APP695 Cells

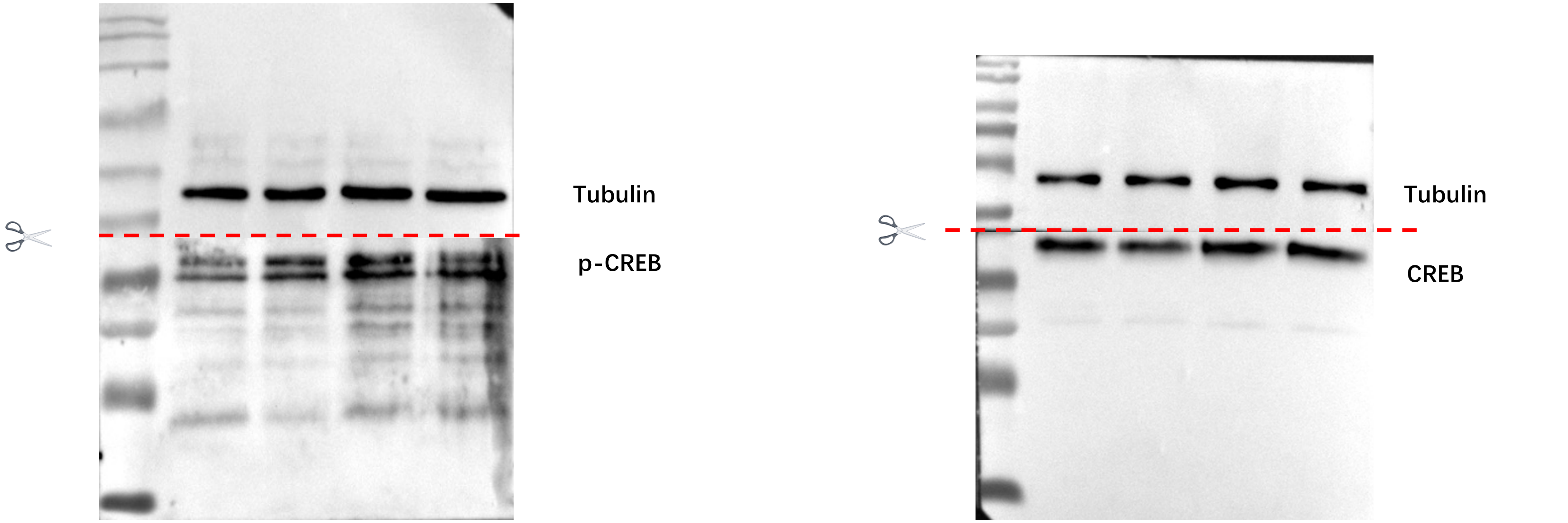

Figure 7A-5

Section S2.1 Raw membranes of Western blot with crop points

Section S2. The Effect of MsA on N2a/APP695 Cells

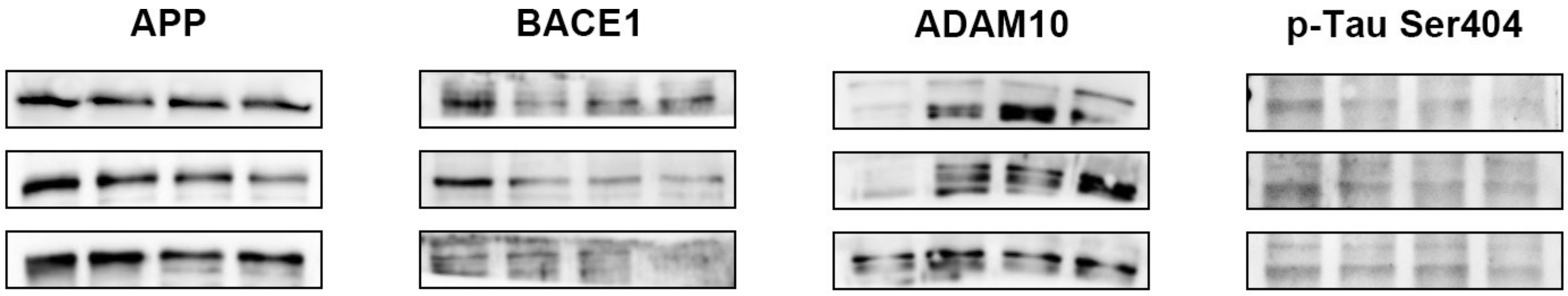

Section S2.2 Replicates employed in the Western Blotting analysis

Section S2. The Effect of MsA on N2a/APP695 Cells

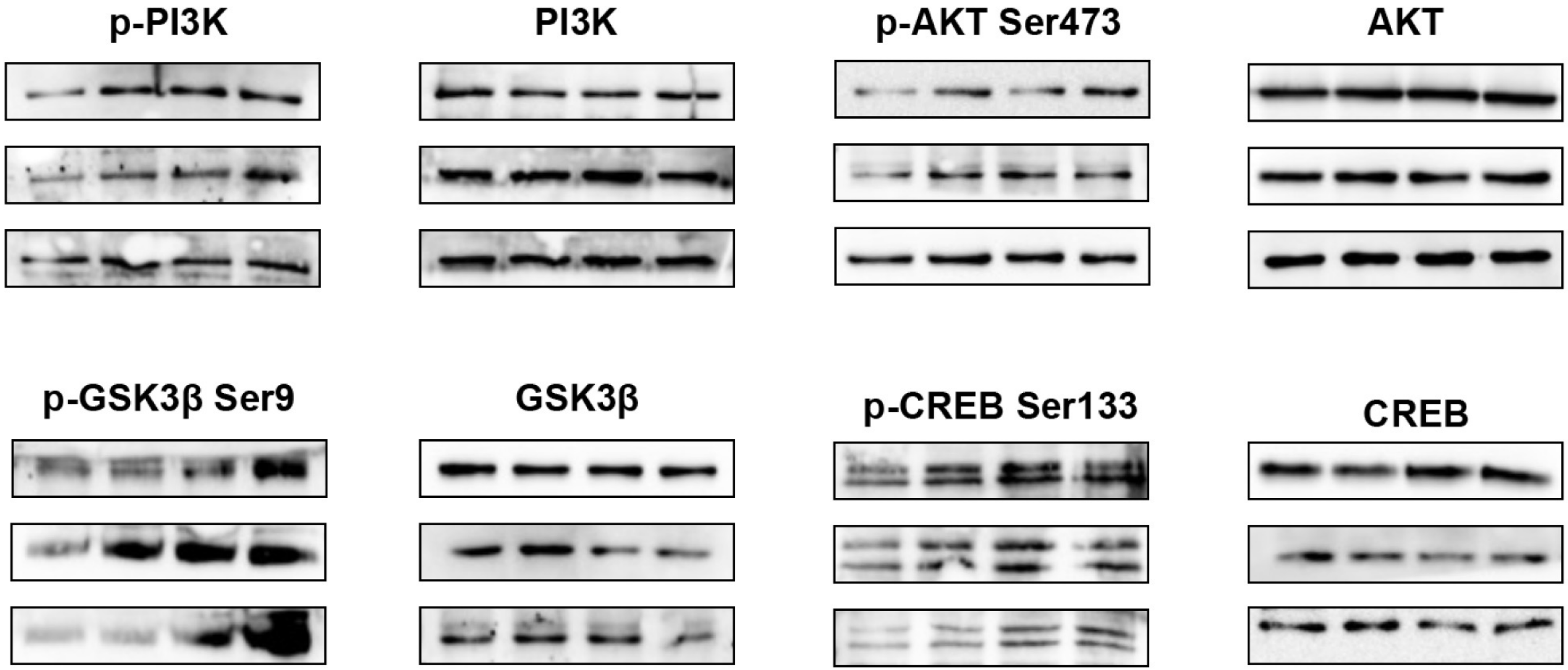

Section S2.2 Replicates employed in the Western Blotting analysis
